# Complement, coagulation and NET pathway activation underlies early host response to blowfly (*Lucilia cuprina*) infestation

**DOI:** 10.1186/s13071-026-07468-0

**Published:** 2026-07-10

**Authors:** Sugandhika Gothami Welikadage, Ying Ting Yang, Shilpa Kapoor, Ching-Seng Ang, Clare A. Anstead, Jean-Pierre Y. Scheerlinck, Trent Perry, Vernon M. Bowles

**Affiliations:** 1 Department of Veterinary Biosciences, Melbourne Veterinary School, Faculty of Science, The University of Melbourne, Parkville, VIC 3010 Australia; 2 Bio21 Molecular Science and Biotechnology Institute, Faculty of Science, The University of Melbourne, Parkville, VIC 3010 Australia; 3 Mass Spectrometry and Proteomic Facility, Faculty of Science, The University of Melbourne, Parkville, VIC 3010 Australia

**Keywords:** Proteomic analysis, Host proteins, Myiasis, Breech strike resistance

## Abstract

**Background:**

Blowfly strike (myiasis), caused mainly by *Lucilia* spp., poses a serious animal welfare concern and results in substantial economic losses to the global sheep industry. However, the early molecular and proteomic responses of sheep skin to larval infestation are not well characterised.

**Methods:**

The early proteomic response in sheep skin following infestation by blowfly *Lucilia cuprina* larvae was assessed in 14 Merino ewes, 7 from a group selected for breech strike resistance and 7 from a non-selected group. Control sites were also established using mock dental plugs without blowfly eggs. After ~ 22 h post-egg implantation, larvae were removed to collect excretory–secretory (ES) products. Skin washings were also collected to recover sheep proteins secreted in response to larval activity. Using liquid chromatography-tandem mass spectrometry (LC–MS/MS), comparisons were made between (1) larval-challenged and mock control sites to identify sheep proteins induced by larval activity and (2) resistant and non-selected sheep. While this paper focuses mainly on the sheep proteins produced in response to larval activity, excretory–secretory (ES) proteins from the larvae were also detected.

**Results:**

In total, 105 proteins were significantly enriched at larval-challenged sites. Following challenge, Gene Ontology (GO) and Kyoto Encyclopedia of Genes and Genomes (KEGG) pathway analyses revealed strong enrichment of the complement and coagulation cascades as well as the neutrophil extracellular trap (NET) formation pathway. Protein–protein interaction analysis identified a major cluster with alpha-2-macroglobulin (A2M) as the seed protein, while the top 10 hub proteins included A2M, serine protease inhibitors (Alfa-1 antitrypsin, Antithrombin III), fibrinogens and plasma proteins such as albumin and haptoglobin. No proteins in the skin exudate differed significantly between resistant and non-selected sheep, and larval ES products also showed no significant differences between these groups.

**Conclusions:**

Early infestation by *Lucilia cuprina* larvae induces a strong, non-specific proteomic response in sheep skin, characterised by activation of the complement and coagulation cascades and NET formation. However, findings in this study suggests that breech strike resistance is unlikely to be associated with differences in skin protein expression during a short infestation.

**Graphical Abstract:**

**Figure:**
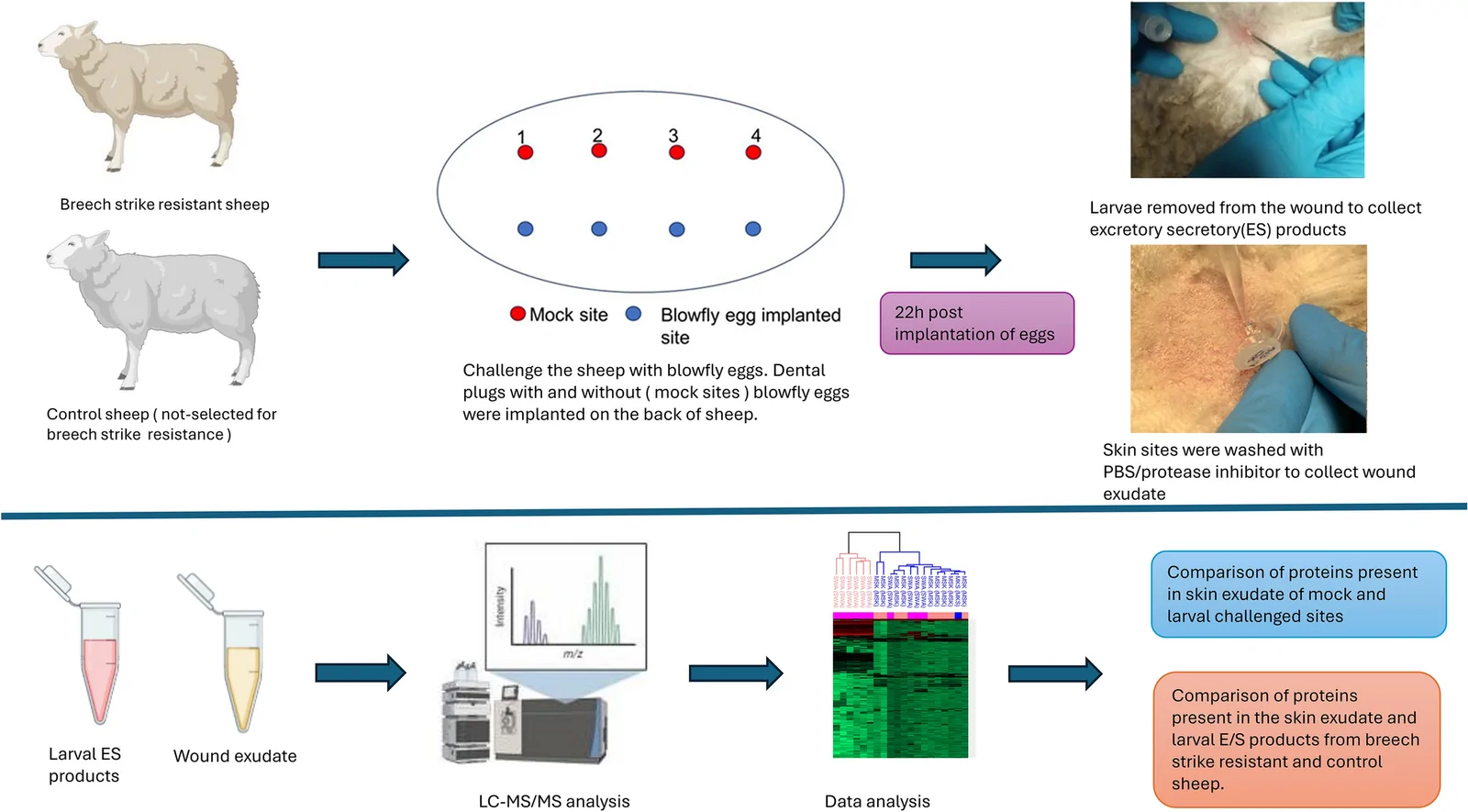

**Supplementary Information:**

The online version contains supplementary material available at https://doi.org/10.1186/s13071-026-07468-0.

## Background

Sheep blowfly strike (ovine cutaneous myiasis) is a major ectoparasitic disease affecting sheep producing countries worldwide, including Australia [1], New Zealand [2], the United Kingdom [3, 4], South Africa [5] and Europe [6, 7]. The infestation is caused primarily by two closely related blowfly species, *Lucilia cuprina* and *L. sericata* [8–10]. In Australia, the subspecies *L. c. dorsalis* (Robineau-Desvoidy) is responsible for approximately 90% of flystrike cases [11–14]. The larvae feed on the host’s skin and exudates, producing large oedematous wounds that can progress to severe systemic infections, sepsis and, in extreme cases, death [15, 16]. The economic burden of blowfly strike is estimated at approximately AUD $323 million annually, through direct stock losses and expenses associated with treatment and preventative measures [17].

Current control strategies rely heavily on chemical insecticides; however, chemical control alone is not sufficient as blowfly has developed resistance to insecticides. Thus, sustainable and effective management of myiasis requires an integrated approach [11, 18, 19]. Such strategies combine chemical, biological and mechanical control methods, including husbandry practices that reduce host susceptibility [20–22]. In addition, genetic approaches, including sterile insect techniques for population suppression and selective breeding of resistant sheep lines, are being explored to achieve sustainable long-term control [23, 24]. Despite these measures, flystrike remains a persistent challenge, highlighting the urgent need for improved understanding of host–parasite interactions and the development of novel control strategies.

*Lucilia* flies are attracted to volatile odour cues emitted by sheep and oviposit on the skin, where newly hatched larvae use their mouthparts and release excretory–secretory (ES) products to initiate an inflammatory response [4, 25], which is characterised by strong cellular and cytokine responses [26–30]. The resulting tissue damage facilitates exudation, which in turn supports the survival and development of first-instar larvae [31]. As larvae develop into the second instar, tissue damage intensifies [25]. However, the influence of host tissue fluids on larval development remains unresolved. Early studies by O’Donell (1980) demonstrated that the anti-inflammatory agent betamethasone suppressed larval activity, suggesting that inflammation is critical for larval establishment [32]. By contrast, Bowles (1990) reported that two major protease inhibitors present in sheep wound exudates inhibited larval growth *in vitro*, indicating that host-derived factors may also contribute to limit the infestation [33].

Previous studies have shown a link between immunological parameters and flystrike resistance, particularly through work on the Trangie fleece-rot resistant sheep flock [34]. Resistant sheep exhibited larger skin wheal reactions to blowfly larval ES products and differential protein expression compared with susceptible animals [34]. Notably, proteins, such as complement 3, α1-antitrypsin and α1-glycoprotein, showed significantly different expression between resistant and susceptible groups, suggesting that host protein responses may contribute to resistance [35].

The current study aimed to characterise the early host response in flystrike lesions using a proteomic-based approach, focusing on proteins expressed during the earliest stages of interaction with blowfly larvae. In addition, breech strike resistant sheep were studied and compared with control sheep that had not been selected for flystrike resistance [36]. The aim of this comparison was to identify any protein-correlates of resistance during the first instar stage, when larvae are most vulnerable and host responses are predicted to be most effective in disrupting larval development [37].

## Methods

### Animals and housing

Fourteen 3-year-old Merino ewes were used in this study, comprising two groups: seven ewes selectively bred for breech strike resistance (R) and seven control ewes, from the same breed. Non-selected (NS) control sheep group are, in theory, likely to be as susceptible as the normal sheep population as they have not been selected for resistance to flystrike in any way (i.e. wild-type). All animals were sourced from the CSIRO Chiswick research facility in Armidale, New South Wales, Australia (30°35′ 18.24″ S, 151°36′ 13.68″ E) [36]. The Armidale breech strike resistant flock was established as part of the Australian Wool Innovation (AWI) and The Commonwealth Scientific and Industrial Research Organisation’s (CSIRO) Breeding for Breech Strike Resistance program (2005–2015), to investigate genetic resistance to breech flystrike under natural field conditions. Sheep were monitored in the field, and breech strikes were recorded through regular flock inspections. In addition to direct flystrike observations, several indicator traits associated with breech strike susceptibility (breech wrinkle, dag score, urine stain, and breech cover) were assessed [36]. These phenotypic records, together with pedigree information, were used to estimate breeding values and guide the selection of animals to generate breech strike resistant sheep flock. Upon arrival at The University of Melbourne animal facility (Parkville), the sheep were given a 2-week acclimatization period prior to experimental procedures. During this time, they were fed a diet of pellets (Rumevite, Ridley AgriProducts, Melbourne, Australia) and lucerne hay and provided ad libitum access to water. Daily monitoring was conducted to assess general appearance, behaviour, appetite and gait.

### Larval challenge

**Fig. 1:**
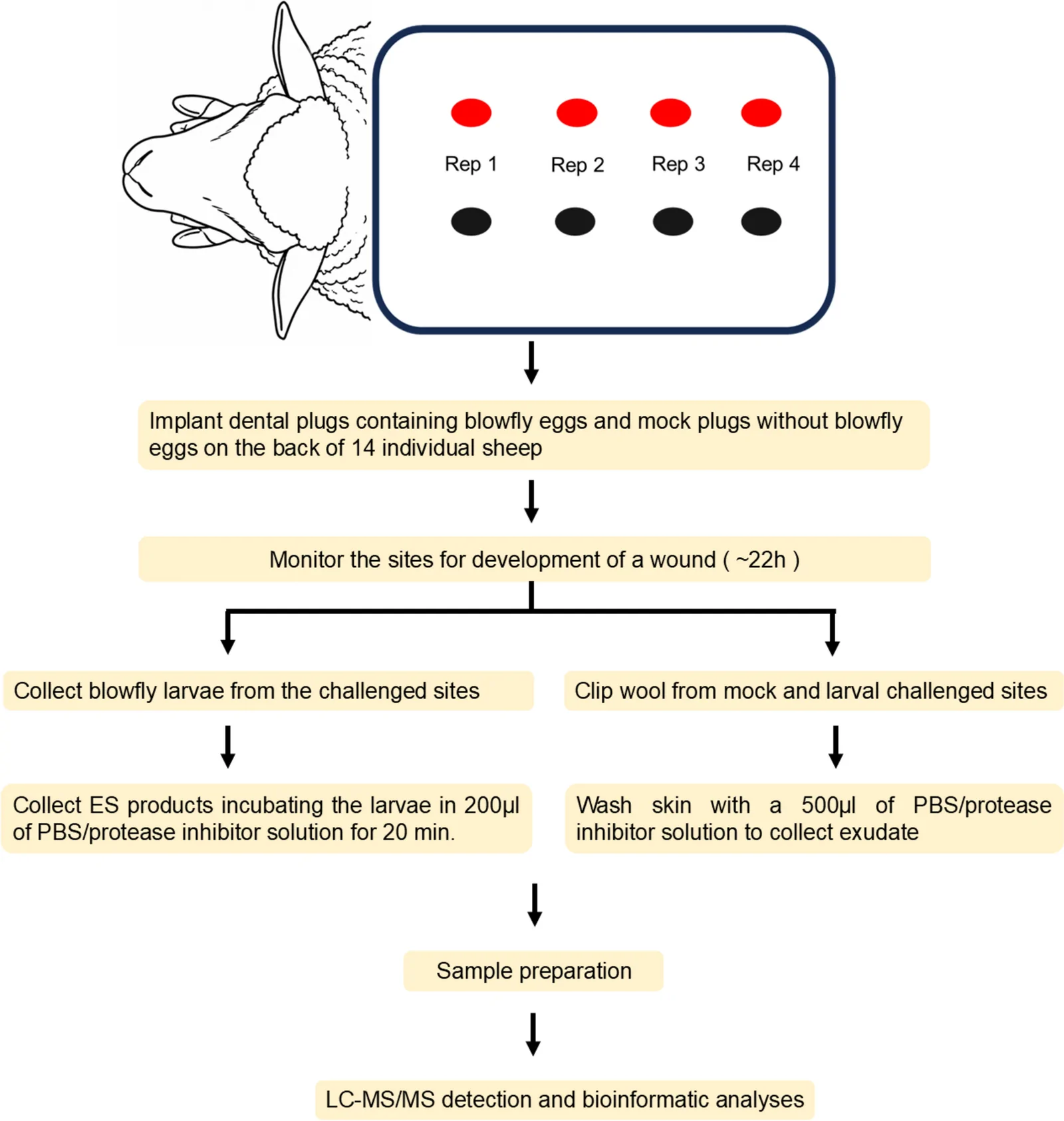
Schematic presentation of the implantation protocol. Dental plugs were placed on the back of each sheep (*n* = 14; 7 breech-strike resistant (R), 7 non-selected (NS) sheep) containing either *Lucilia cuprina* eggs (infested) or no eggs (control). Four replicate sites were used per sheep. Sites were monitored for wound initiation after which blowfly larvae were collected into labelled tubes. The fleece surrounding each site was clipped and the sites were washed with PBS supplemented with protease inhibitors (cOmplete™, EDTA-free Protease Inhibitor Cocktail, Sigma-Aldrich, Merck KGaA, Darmstadt, Germany). Wash fluids were collected for proteomic analysis of host-derived proteins associated with the early stages of flystrike lesion development. PBS, phosphate-buffered saline

Eggs of *Lucilia cuprina dorsalis* were obtained from a laboratory-susceptible (LS) strain maintained at the Bio21 Molecular Science and Biotechnology Institute Insectary (The University of Melbourne). This LS strain was originally established and maintained for more than 20 years in the laboratory of Peter James at The University of Queensland, Australia [38]. The colony was reared using an established culture method [39], with bovine liver provided as a substrate for oviposition and larval development. The LS strain has subsequently been maintained for more than 3 years in the insectary at Bio21 Institute. Adult flies were kept at room temperature (RT) in cages supplied with water and protein powder (Sustagen, Nestlé Health Science, Germany). Diced beef was provided to stimulate ovarian development and promote oviposition with eggs collected directly from the meat substrate. Each sheep received eight implantation sites along the dorsum: four experimental sites containing blowfly eggs placed on moist cotton dental plugs and four mock-control sites with dental plugs only (Fig. 1) [implantation protocol adapted from [34]].

On day 1, eggs (~ 30 mg) were weighed and placed on cotton dental plugs pre-soaked in distilled water. Sheep skin was prepared by parting the wool and wetting the fleece with ~ 200 ml of water. Implants were placed on the skin, and the surrounding wool was gathered together and held in place with a rubber band. Under these conditions, egg hatching was expected within 15–20 h [40]. At ~ 22 h post-implantation, the rubber band was removed and the fleece around experimental and control sites was clipped using Oster clippers (Sunbeam-Oster Co. Inc., Florida, USA). Exudates were collected by washing each site with 500 μl phosphate-buffered saline (PBS) containing protease inhibitor (cOmplete™, EDTA-free Protease Inhibitor Cocktail, Sigma-Aldrich, Merck KGaA, Darmstadt, Germany) and samples were recovered into 1.5 ml Eppendorf tubes. Mock sites were also processed as described for the challenged sites. Larvae raised on R sheep and NS sheep were also collected into 1.5 ml Eppendorf tubes (Eppendorf, Hamburg, Germany), with 50 larvae in each tube and incubated at RT for 20 min in PBS/protease inhibitor solution to collect larval ES products.

### Protein sample preparation for mass spectrometry analysis

Skin washings from each site and larval ES products were snap-frozen in liquid nitrogen and stored at −80 °C. Prior to processing, protein concentration was measured using a BCA assay (Pierce BCA assay Kit, Thermo Fisher scientific, Massachusetts, USA).

Samples were processed using a modified single-pot digestion [41]. Each sample (80 µl) containing 1 µg of protein was reduced with 4 µl of 100 mM dithiotreitol (DTT) for 20 min at 60 °C mixing at 1000 rpm in a thermomixer (Eppendorf, Hamburg, Germany). The solution was allowed to cool at RT and alkylated by adding 12 µl of 100 mM Iodoacetamide (IAA) in 25 mM triethylammonium bicarbonate (TEAB) in the dark for 20 min. DTT (5 µl of 100 mM) was added and incubated for a further 10 min at RT. This was followed by adding 2 µl of Sera-Mag/SP3 beads (Sigma-Aldrich, Merck KGaA, Darmstadt, Germany) from 50 µg/µl stock to the sample and mixed by pipetting to ensure homogenisation of SP3 beads. To induce binding, the same volume of 80% ethanol (103 µl) was used and incubated at 24 °C for 5 min mixing at 1000 rpm in a thermomixer. Tubes (1.5 ml Eppendorf) were placed in a magnetic rack and incubated until beads migrated to the tube wall. The unbound supernatant was discarded and 180 µl of 80% ethanol was again added to the tubes. Tubes were again placed in the magnetic rack and incubated until beads were against the wall of the tube. This process was repeated twice, extracting as much ethanol as possible on the last rinse. Protein aggregates were digested by the addition of 100 µl digestion solution (Trypsin + LysC mix 2:25 enzyme:protein ratio in 100 mM of ammonium bicarbonate; Promega, Madison, USA). The mixture was sonicated for 30 s to disaggregate the beads then incubated at 37 °C for 18 h at 1000 rpm in the thermomixer. The next morning, the sample was spun down at 20,000 *g* at 24 °C for 1 min, and the supernatant recovered and transferred to a fresh 1.5 mL Eppendorf tube. After digestion, peptides were acidified with 2% acetonitrile and 0.05% trifluoroacetic acid (TFA) before liquid chromatography-tandem mass spectrometry (LC–MS/MS) analysis at the Bio21 Mass spectrometry and Proteomic Facility.

### MS analysis

Liquid chromatography-tandem mass spectrometry (LC–MS/MS) analysis was performed either on an Orbitrap Q Exactive plus or Exploris 480 Mass Spectrometer (ThermoFisher Scientific Massachusetts, USA) as described previously [42]. Instrument selection was based on availability at the time of each experiment. To ensure consistency, all samples within a given experiment were analysed on the same mass spectrometer, while different instruments were used across separate experiments as required by availability. The liquid chromatography (LC) system was equipped with an Acclaim Pepmap nano-trap column (Dionex-C18, 100 Å, 75 μm × 2 cm) and an Acclaim Pepmap RSLC analytical column (Dionex-C18, 100 Å, 75 μm × 50 cm). Tryptic peptides were injected into the enrichment column at an isocratic flow of 5 μl/min of 2% (*v*/v) acetonitrile containing 0.1% (*v*/v) formic acid for 6 min applied before the enrichment column was switched in line with the analytical column. The eluents were 0.1% (v/v) formic acid, 5% dimethyl sulfoxide (DMSO) (solvent A) in water and 100% (v/v) acetonitrile, 5% DMSO and 0.1% (v/v) formic acid (solvent B). The flow gradient was (1) 0–6 min at 2% B; (2) 6–40 min at 3–25% B; (3) 40–48 min at 25–45% B; (4) 48–50 min at 40–80% B; (5) 50–53 min at 80–80% B; (6) 53–54 min at 80–3% and equilibrated at 2% B for 10 min before injecting the next sample.

For the data-dependent acquisition (DDA) experiments on an Orbitrap Exploris 480, the mass spectrometer was operated in the DDA mode, whereby full MS1 spectra were acquired in a positive mode at 60,000 resolutions using Tune (ver. 2.0.182.25). The ‘top speed’ acquisition mode with 3 s cycle time on the most intense precursor ion was used, whereby ions with charge states of 2–7 were selected. Tandem mass spectrometry (MS/MS) analyses were performed by 1.6 m/*z* isolation with the quadrupole, fragmented by higher energy C-trap dissociation (HCD) with a collision energy of 30%. MS2 resolution was at 15,000. Dynamic exclusion was activated for 30 s. Automatic Gain Control (AGC) target was set to standard with auto maximum injection mode.

For the DDA experiment on the Orbitrap Q Exactive plus, the mass spectrometer was operated in the DDA mode, whereby full MS1 spectra were acquired in positive mode, 70,000 resolution, AGC target of 3 × 10^6^ and maximum IT time of 50 ms. Fifteen of the most intense peptide ions with charge states ≥ 2 and an intensity threshold of 1.7 × 10^4^ were isolated for MS/MS. The isolation window was set at 1.2 m/*z* and precursors fragmented using normalized collision energy of 30, a resolution of 17,500, AGC target of 1 × 10^5^ and maximum IT time of 100 ms. Dynamic exclusion was set at 30 s.

For the DDA experiment on the Orbitrap Lumos, the mass spectrometer operated in DDA mode, scanning from 300 to 1600 m/*z* at a resolution of 60,000. High-Field Asymmetric Waveform Ion Mobility Spectrometry (FAIMS) separations were conducted with the following parameters: inner and outer electrode temperatures set to 100 °C, FAIMS carrier gas flow at 4.7 L/min and nitrogen (N_2_) as the carrier gas. The cycle time in ‘top speed acquisition’ mode was 3 s for a single compensation voltage (CV) experiment, while experiments using two CVs (−40 V and −60 V) had a cycle time of 1.5 s each. MS/MS analyses were performed with a quadrupole isolation width of 1.6 m/*z*, fragmentation by collision-induced dissociation (CID) at 35% collision energy, an activation time of 10 ms and an activation Q of 0.25. Fragment ion analysis was carried out in the ion trap using the ‘Turbo’ speed scanning mode, with dynamic exclusion enabled for 30 s.

### Database search and bioinformatics analysis

Peptide sequencing results were processed using the MaxQuant search engine using default search parameters [43]. The *Ovis aries* (Reference Proteome ID: UP000002356), data from the Universal Protein Resource (UniProt) database and the blowfly reference proteome (GCA_025217265.1) were used for peptide identification [44, 45]. A BLAST search against FlyBase (https://flybase.org/) and the NCBI database was performed to further match the peptide sequences [46, 47]. Trypsin/P was set as an enzyme allowing up to two missed cleavages; the carbamidomethylation of cysteine was used as fixed modification; N-terminal protein acetylation and methionine oxidation were applied as variable modifications; the minimum and maximum peptide length was set to 8 and 25 amino acids, respectively. Precursor ion tolerance was set at 20 and 10 ppm in the first and the main peptide search, respectively, whereas the mass tolerance for MS/MS fragment ions was set at 40 ppm. Peptide spectrum match (PSM) and protein identifications were filtered using a target-decoy approach at a false discovery rate (FDR) of 1%. Label-free quantification (LFQ) of proteins was carried out using the algorithm integrated into MaxQuant with the minimum ratio count set at 2 [43]. The MaxQuant generated results file was then entered into Perseus (v 1.6.15.0) for further analysis [48]. The data were filtered to eliminate hits to the reverse database and proteins only identified with modified peptides. The ‘removing contaminant sequence’ filter was not applied during the search, to not exclude any sequences. In addition, only proteins observed in at least two replicate samples were subjected to data processing. Filtered data were log_2_ transformed. When more than one protein was listed in the peptide identification column only the first listed was used in the analysis [49]. Two independent comparisons were performed using the Student’s *t*-test to analyse/compare protein expression and secretion patterns between experimental groups.

Of the 14 sheep sampled, 4 were used for mock versus larval challenge site comparisons, and 8 were used for comparisons between resistant (R) and non-selected (NS) sheep. Samples from two sheep were excluded from the analyses owing to poor sample quality.

### Comparison of mock and larval-challenged sites

To identify proteins produced by sheep in response to blowfly larval activity, as well as blowfly-derived proteins released onto the sheep’s skin during host–parasite interactions, mock and larval-challenged sites were compared. Ten samples from mock sites of four sheep (two R and two NS) were compared with ten samples from larval-challenged sites on the same four sheep. Missing values were replaced using default imputation settings in Perseus to enable downstream statistical comparisons.

### Comparison of skin exudate from resistant and non-selected sheep

Proteomic profiles of skin exudates from R and NS sheep were compared using samples collected from four R and four NS animals, each represented by three replicates.

### Comparison of larval ES products from resistant and non-selected sheep

To determine whether larvae secrete proteins differently when feeding on resistant versus non-selected sheep, ten ES samples collected from larvae fed on four R sheep and four NS sheep were compared. Each comparison was conducted using the Student’s *t*-test with false discovery rate (FDR) permutation to identify significant differences in protein expression or secretion patterns. The S0 parameter and corrected *P*-value were set to 0.1 and *P* < 0.05, respectively. Differentially expressed proteins were visualized using volcano plots generated in GraphPad Prism software (v 9.5.0) with the *x*- and *y*-axes representing log_2_ fold change and −log_10_ *P*-value, respectively. The proteins that showed differences in the fold-change (Fc) > 2 and a corrected *P* < 0.05 were considered significantly differentially produced.

### Differentially produced proteins enrichment analysis

To analyse Gene Ontology (GO) components, differentially expressed proteins (molecular functions, cellular components and biological processes) were searched on UniProt/FlyBase databases and then using The Database for Annotation, Visualization and Integrated Discovery (DAVID) [44, 46, 50, 51].

### PPI network analysis

Protein–protein interaction (PPI) network analysis was performed using the Search Tool for the Retrieval of Interacting Genes (STRING) (https://string-db.org/) online tool to assess the correlation between two or more proteins using default parameters [52]. The PPI among the top 10 hub proteins was further calculated by cytoHubba plugins [53] in Cytoscape software (v 3.9.1). Additionally, molecular complex detection (MCODE) [54] plugin to screen highly interconnected gene clusters in the PPI network was used. Within each cluster, the protein exhibiting the greatest degree of interconnectivity was designated as the seed protein. Clusters were ranked on the basis of their related scores, which was obtained by the interconnection determination. The prediction of clusters is based on vertex weighting by local neighbourhood density and outward traversal from a locally dense seed protein to isolate the dense regions according to given parameters. The criteria for selection in MCODE were as follows: MCODE scores ≥ 4, degree cutoff = 2, node score cutoff = 0.2, k-core = 2 and max depth = 100.

## Results

### Comparison of proteins in skin washings of mock and larval-challenged sites

**Fig. 2:**
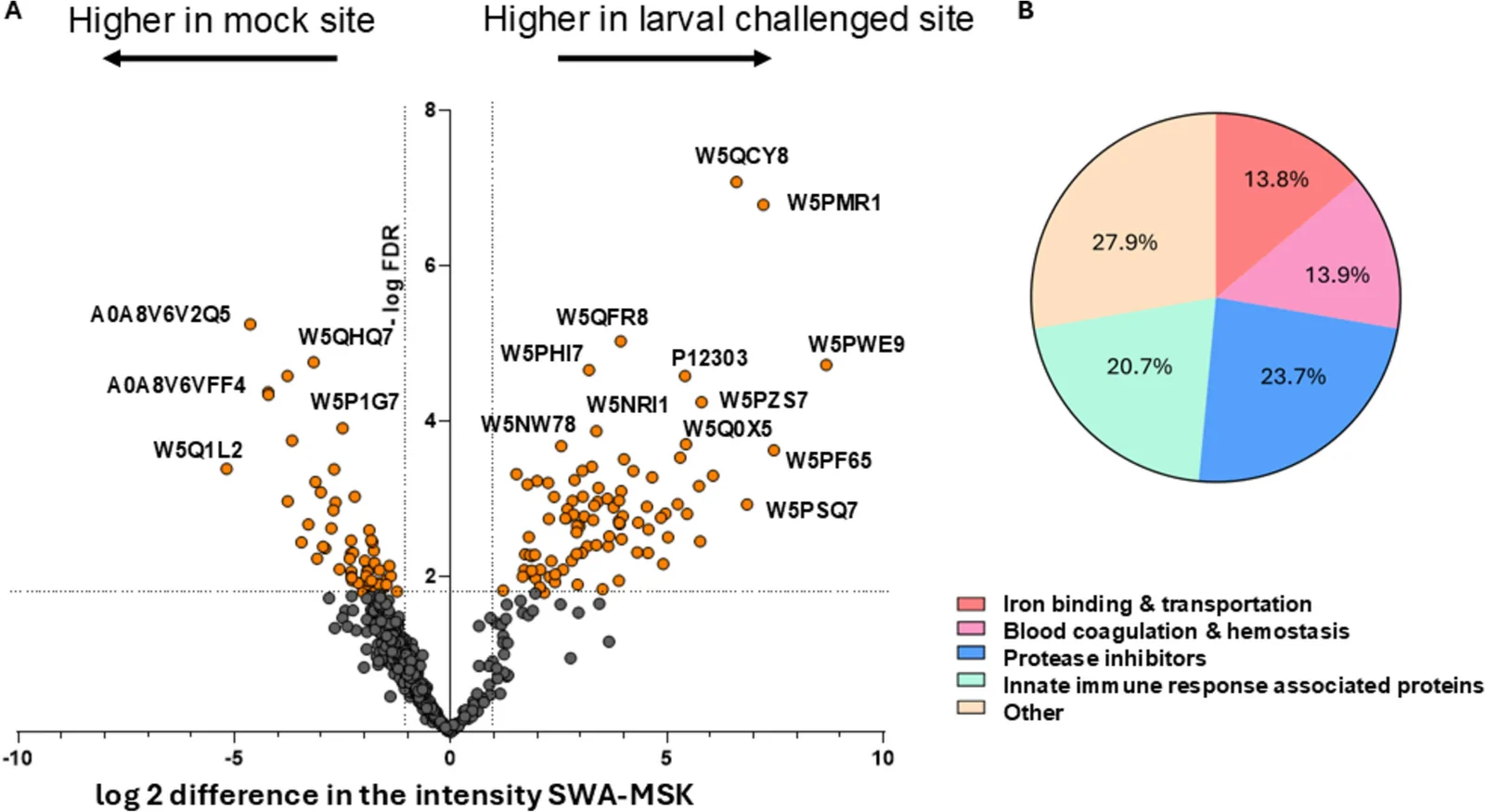
Sheep proteins significantly elevated in larval infested sites. **A** A volcano plot comparing the proteins present in mock sites and larval-challenged sites. The difference in the intensity of each protein between the skin exudate samples (SWA) and mock skin washing samples (MSK) was calculated by Perseus, and the significant difference was evaluated by Student’s *t*-test. The analysis was performed with fold change of 2 and a false discovery rate (FDR) of < 0.05. Out of the 132 differentially expressed proteins, 105 were upregulated. Some of the sheep-derived proteins that show a higher fold change and higher significance are labelled with their UniProt identification numbers. **B** The percentage of sheep-derived proteins involved in different functional categories based on the Gene Ontology (GO) of molecular function

The compositions of proteins of skin washings/exudate collected from larvae-infested sites were compared with skin washings collected from the mock sites using mass spectrometry. A total of 526 sheep proteins and 148 blowfly proteins were detected in at least one sample out of the 20 samples tested (Fig. 2A). Exudate comprised both larval proteins and proteins produced by the sheep in response to the interaction with blowfly larvae. Comparing proteins present in mock and larval-challenged sites identified 132 significantly different proteins (Fig. 2A). The concentration of a total of 105 sheep proteins out of 132, were significantly higher (two sample *t*-test, *P* < 0.05) in the larval challenged sites compared with mock sites (Additional file 1: Table S1). On the basis of the molecular function, inflammatory and immune response-related proteins were classified and represented in the pie chart (Fig. 2B, Additional file 2: Table S2). Interestingly, 23% of these proteins are involved in protease inhibition, while 14% showed anticoagulant properties. Overall, 21% are involved in innate immune response and antibacterial defence, while 13% have an association with ion binding and transportation. Most of the proteins show additional functions including extracellular matrix formation, cell adhesion and actin filament binding (Fig. 2B).

### GO and KEGG pathway analysis of differentially expressed proteins

**Fig. 3:**
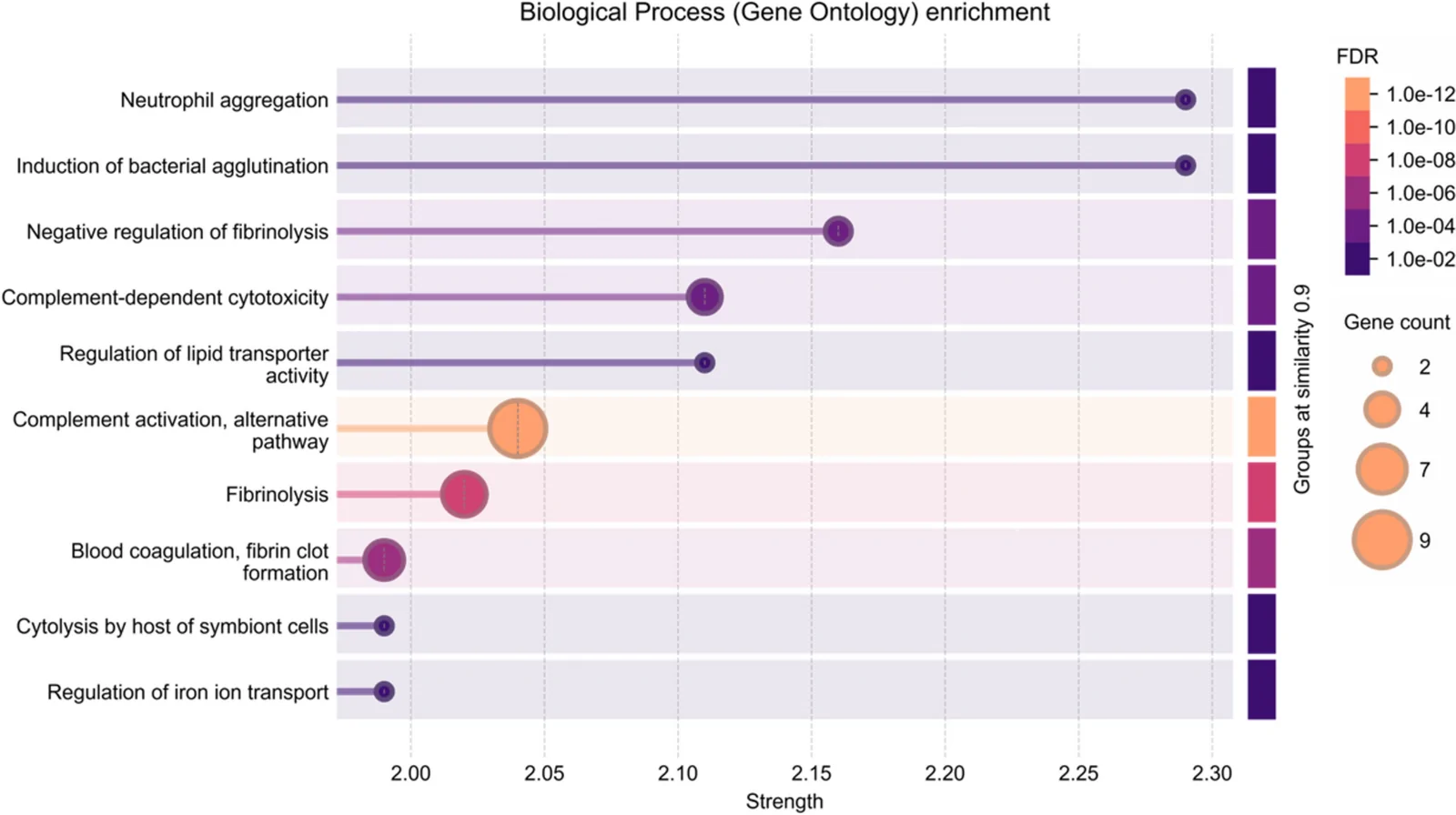
A bubble plot of Gene Ontology (GO) biological processes of the sheep proteins. GO analysis of sheep proteins present at significantly higher levels in skin exudate samples collected from *Lucilia*-infested sites compared with mock sites. Fisher’s exact test with a false discovery rate (FDR) < 0.05. Size of the bubble represents number of genes/proteins involved in each pathway. Plot was generated in the STRING database

Proteins collected from sheep skin exudates were categorised into different enrichment clusters using the information extracted from the DAVID and STRING databases using sheep whole genome as the background (GCF_016772045.2). Enrichment scores for each cluster were then recorded on the basis of the biological process. Highly enriched pathways included complement activation and pathways involved in blood coagulation and fibrinolysis (Fig. 3, Additional file 3: Table S3).

**Fig. 4:**
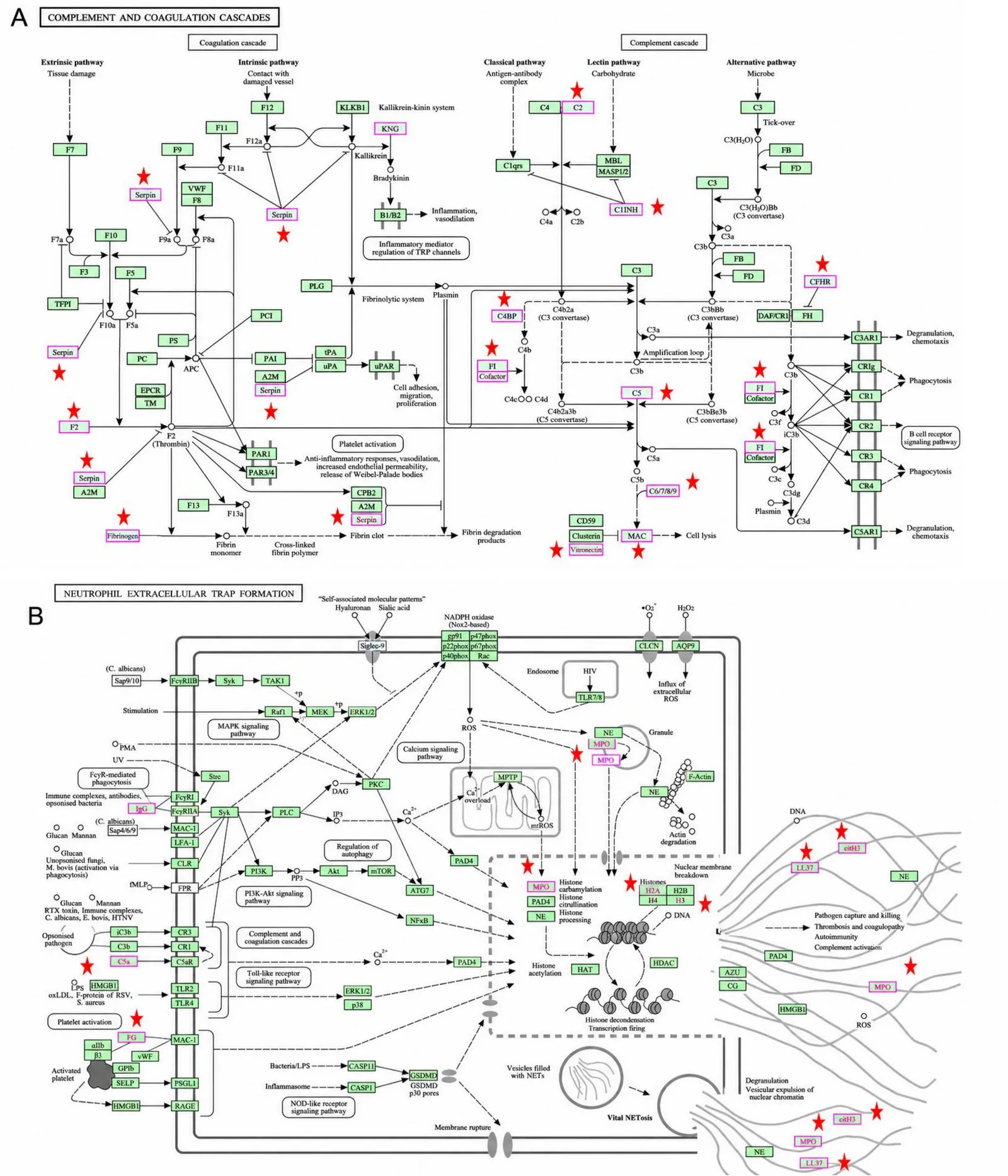
KEGG pathway enrichment analysis of proteins significantly enriched in larval-challenged sites. **A** KEGG pathway map illustrating proteins associated with complement and coagulation cascade. **B** KEGG pathway map illustrating proteins involved in neutrophil trap formation. Red stars indicate significantly enriched proteins detected in exudate samples that are involved in each pathway. Benjamini–Hochberg corrected *P*-value < 0.05

In addition to the GO terms, several significant KEGG pathways (*P* < 0.05) were identified (Additional file 4: Table S4). Notably, complement and coagulation cascade was enriched significantly with a Benjamini–Hochberg corrected *P*-value of 6.85 × 10^–17^ (Fig. 4A) while pathway of neutrophil extracellular trap (NET) formation was highlighted with 1.54 × 10^–9^ (Fig. 4B).

### PPI network analysis

**Fig. 5:**
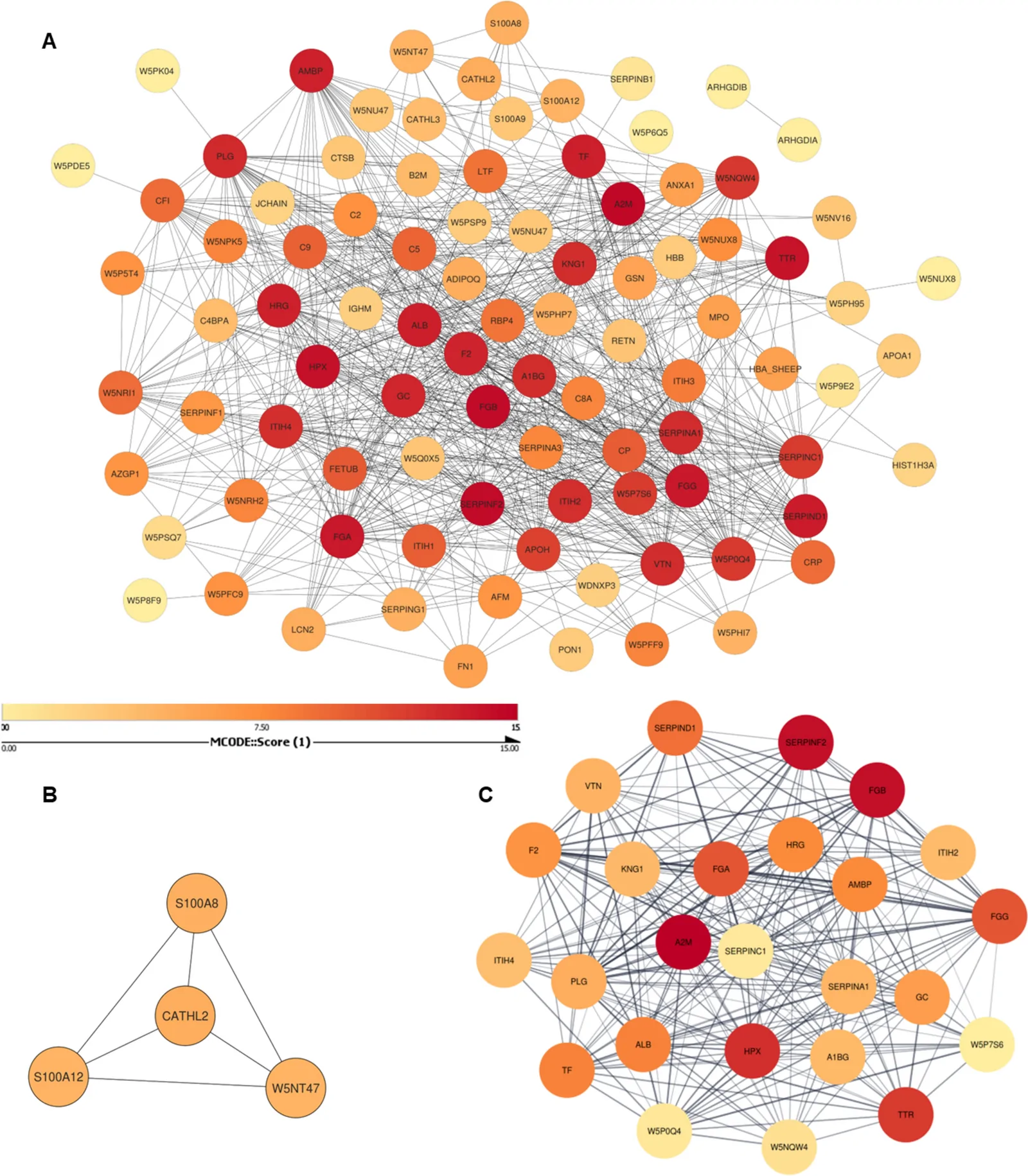
Protein–protein interaction (PPI) network of proteins identified in exudates from larval-infested sheep skin. **A** Global PPI network of exudate proteins from larval-challenged sites. Each node represents a protein, and edges indicate experimentally validated or predicted protein–protein associations. Node colour intensity reflects the MCODE node score, with dark red nodes representing highly connected hub proteins (e.g. A2M, FGB, SERPINF2, HPX, TTR) and yellow nodes representing proteins with fewer interactions. Two major protein clusters were identified using the MCODE cluster analyser. **B** Cluster centred on cathelicidin-2 (CATHL2) as the seed protein. **C** Cluster centred on alpha-2-macroglobulin (A2M) as the seed protein

Protein–protein interactions (PPI) were analysed using the STRING database for all proteins detected in the skin exudates of larval-challenged sites, as well as for proteins significantly enriched in these samples. The resulting PPI network comprised 97 protein nodes and 755 edges. Of the 105 proteins significantly elevated in exudates, eight were not connected to the main network and one protein pair formed an isolated interaction. Cluster analysis using MCODE identified two key protein modules. The first cluster identified with a score of 4 centred on cathelicidin-2 (CATHL2) as the seed protein (Fig. 5B). The second cluster, with a score of 20.3, centred on A2M as the seed protein (Fig. 5C).

**Fig. 6:**
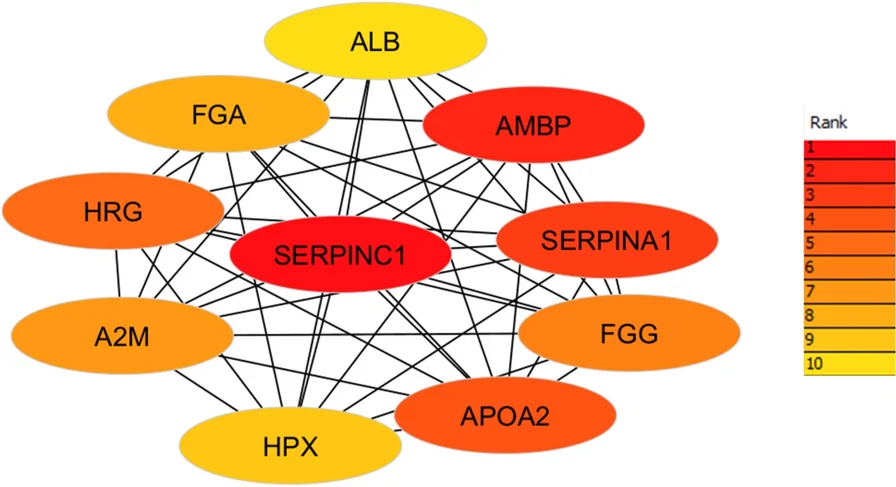
The protein–protein interaction (PPI) network of the top 10 hub proteins. Each node represents a protein and the edges denote protein–protein associations derived from the STRING database. Node colour intensity corresponds to the hub rank: red indicates the highest-ranked (most connected) proteins, while yellow indicates lower-ranked nodes. Figure generated using cytoHubba plugin in Cytoscape software (v 3.10.3)

The top 10 hub proteins (Fig. 6) were selected on the basis of the maximal clique centrality (MCC) method implemented in CytoHubba, a powerful algorithm for identifying hub proteins (most influential nodes in a protein network), that was used to calculate centrality scores on the basis of maximal clique centrality (Table 1).

**Table 1:** List of top 10 hub proteins identified from the protein–protein interaction (PPI) network using the cytoHubba plugin in Cytoscape

Rank	Name	Symbol	Protein name	Score
1	ENSOARP00000013845	SERPINC1	SERPINC1/antithrombin III	2.498 × 10^12^
2	ENSOARP00000006566	AMBP	Protein AMBP	2.493 × 10^12^
3	ENSOARP00000015963	SERPINA1	SERPINA1/Alfa-1 antitrypsin	2.491 × 10^12^
4	ENSOARP00000010518	APOA2	Apolipoprotein A-II	2.488 × 10^12^
5	ENSOARP00000022050	HRG	Histidine-rich glycoprotein	2.486 × 10^12^
6	ENSOARP00000017896	FGG	Fibrinogen	2.483 × 10^12^
7	ENSOARP00000001046	A2M	Alpha-2-macroglobulin	2.482 × 10^12^
8	ENSOARP00000017968	FGA	Fibrinogen alpha chain	2.477 × 10^12^
9	ENSOARP00000019654	HP	Hemopexin	2.315 × 10^12^
10	ENSOARP00000014782	ALB	Albumin	2.11 × 10^12^

### Comparison of skin exudate from resistant and non-selected sheep

**Fig. 7:**
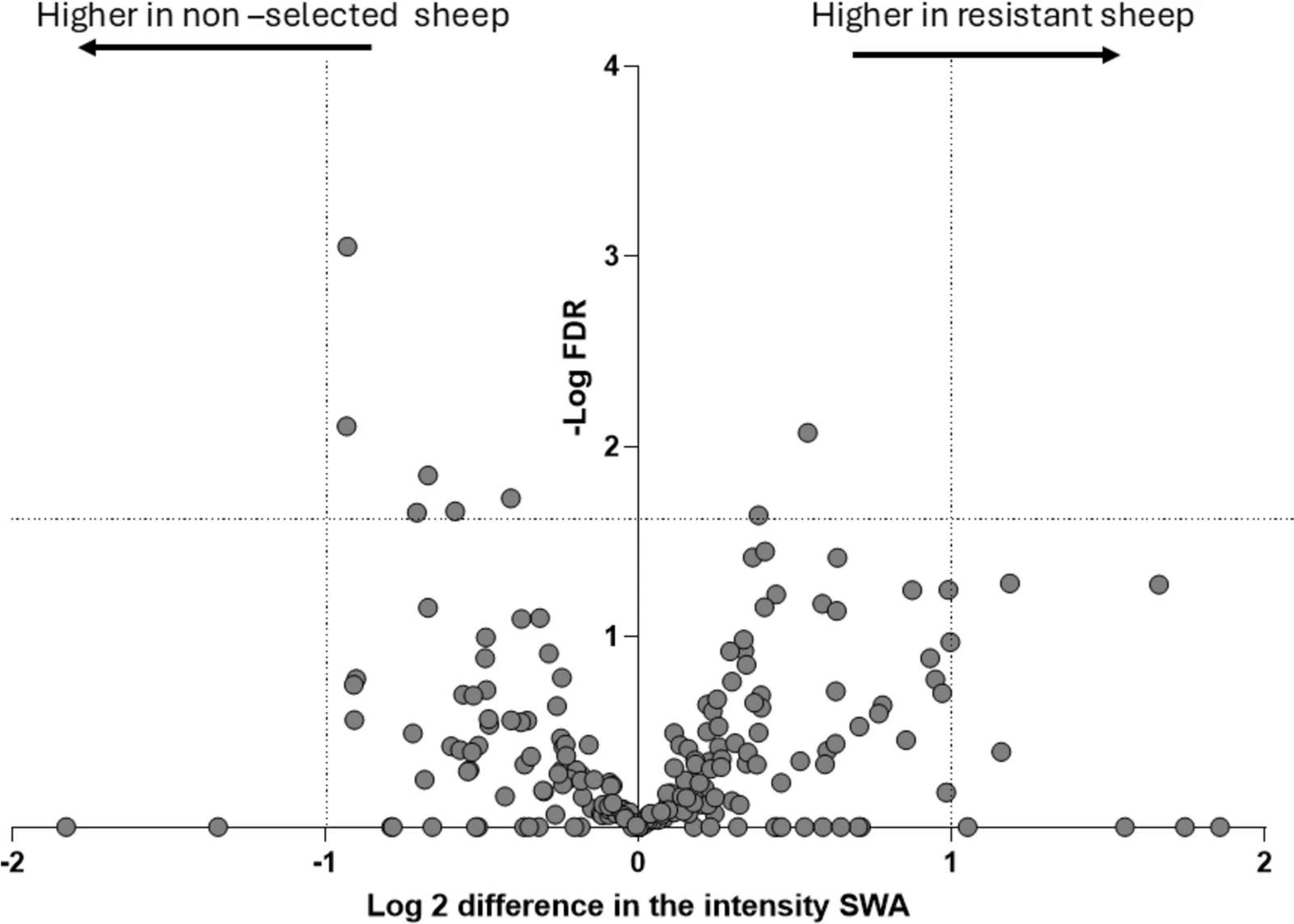
A volcano plot comparing protein abundance between resistant (R) and non-selected (NS) sheep following larval challenge. The plot shows the log_2_ fold change (log Fc) in protein intensity between R and NS sheep skin exudates (*x*-axis) and the corresponding −log_10_ false discovery rate (FDR) values (*y*-axis). Each dot represents an individual protein. Proteins to the right of the vertical axis indicate higher abundance in R sheep, whereas those to the left indicate higher abundance in NS sheep. No proteins showed statistically significant differences (*P* < 0.05, log Fc > 1) between the two groups

A comparative analysis of proteins identified in both the skin washings/exudate of sheep from R and NS groups was performed to investigate the differences in the protein abundance between the two groups in response to larval challenge using Student’s *t*-test (Additional file 5: Table S5). However, no statistically significant differences (*P* < 0.05) were detected between the R and NS groups (Fig. 7). From this analysis, a total of 98 sheep proteins were detected in at least three of the four individuals within either the R or NS group.

### Comparisons of ES products from larvae fed on R and NS sheep

**Fig. 8:**
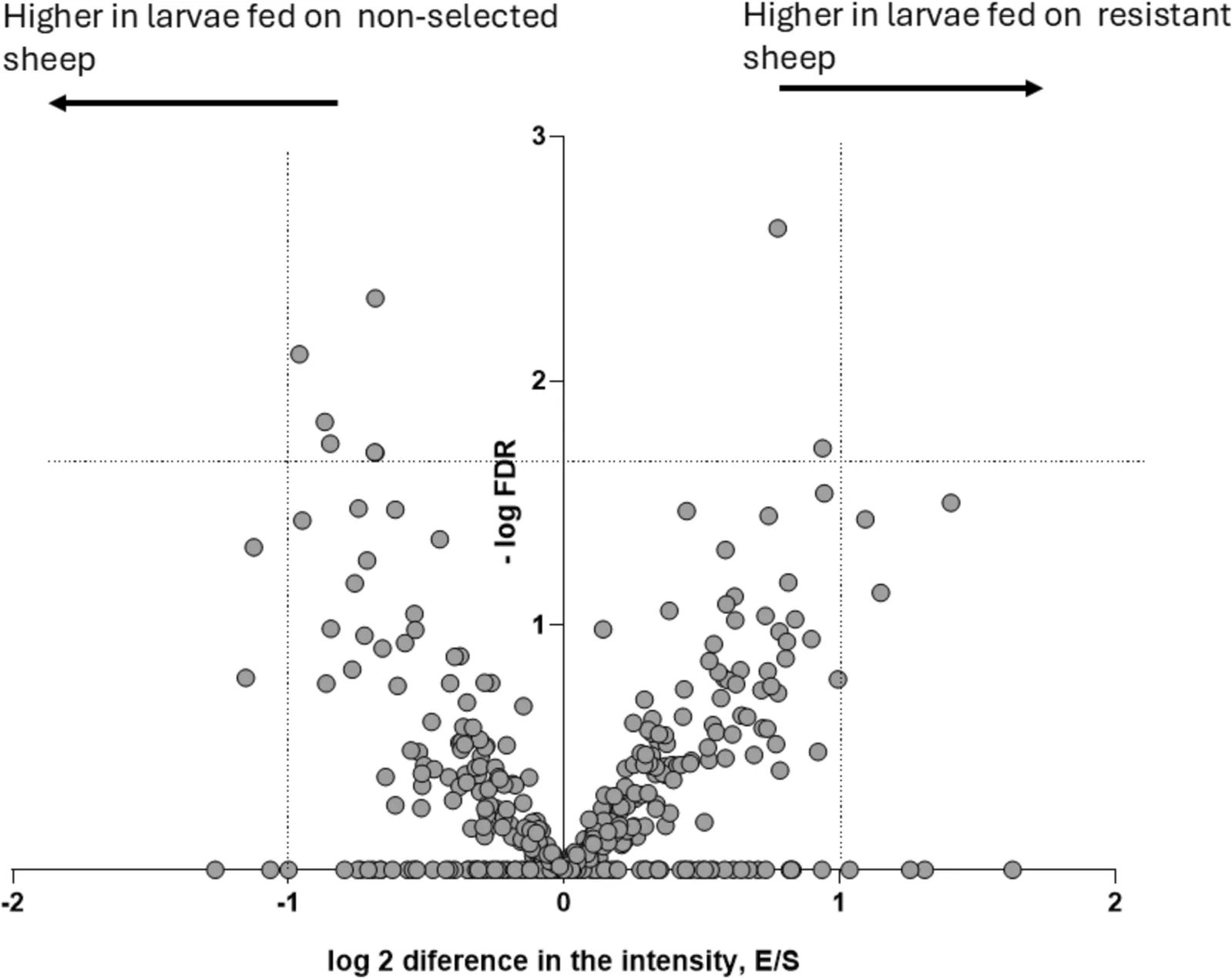
A volcano plot comparing protein abundance of larval ES products collected from larvae fed on resistant (R) and non-selected (NS) sheep. The plot shows the log_2_ fold change (log Fc) in protein intensity between larval ES products (*x*-axis) and the corresponding −log_10_ false discovery rate (FDR) values (*y*-axis). Each dot represents an individual protein. Proteins to the right of the vertical axis indicate higher abundance in larvae fed on R sheep, whereas those to the left indicate higher abundance in larvae fed on NS sheep. No proteins showed statistically significant differences (*P* < 0.05, log Fc > 1) between the two groups

Comparative analysis on ES products collected from larvae fed on R sheep and larvae fed on NS sheep was also carried out to investigate any potential differences in the protein quantity excreted into ES products on the basis of the group of sheep that the larvae fed on. As with previous observations, no difference was identified in ES products collected from larvae fed on the two sheep groups (*P* < 0.05). However, 139 blowfly proteins were found in larval samples collected from at least three sheep tested out of four, while 662 blowfly proteins were detected at least one out of the 20 samples (Fig. 8).

## Discussion

Flystrike, caused by *Lucilia cuprina*, remains a major welfare and economic concern for the sheep industry, yet the early molecular events underlying host–parasite interactions are not well understood [9, 11]. In this study, we employed a proteomics-based approach to investigate protein composition, function and interactions to better understand the molecular mechanisms contributing to flystrike pathogenesis [20, 55–58]. Using this approach, we generated the first comprehensive proteomic characterisation of the early host response to blowfly larval challenge in sheep. By examining dynamic changes in protein expression, particularly in the host response, proteomics offers valuable insights into host–parasite interactions and provide a foundation for identifying potential candidate biomarkers for the genetic selection of resistant sheep [59–62]. Analysis of protein changes in skin exudates from larval-challenged and mock control sites revealed the presence of host-derived serum proteins permeating through the skin, potentially contributing to the early response against larval invasion and tissue injury. Protein–protein interaction network analysis of these proteins detected in the exudate samples further identified two distinct functional clusters representing major components of the early immune response.

The first cluster comprised CATHL2, S100A8, S100A12 and W5NT47. Within this cluster, CATHL2 (cathelicidin-2) emerged as the central hub, interacting with all other proteins. Cathelicidins are primarily recognised as antimicrobial peptides; however, they also modulate neutrophil functions, including the release of chemoattractants and the recruitment of neutrophils to sites of inflammation [63, 64]. S100A8 and S100A12, which interacted with Cathelicidin, are produced by neutrophils, monocytes, macrophages and keratinocytes and are upregulated under inflammatory conditions [65]. These proteins are classified as alarmins or damage-associated molecular patterns (DAMPs), which are endogenous molecules rapidly released following tissue injury. As DAMPs, S100 proteins engage cell surface receptors, such as toll-like receptor 4 (TLR4), receptor for advanced glycation end-products (RAGE) and G-protein-coupled receptors (GPCRs), triggering intracellular inflammatory signalling cascades, promoting the expression of proinflammatory cytokines and contributing to immune regulation [66–70]. Interestingly, elevated levels of S100A12 have previously been reported in cattle infested with *Echinococcus granulosus* [71]. In addition, upregulation of the *S100A12* gene has been observed in sheep infested with *Psoroptes ovis* [72] and in camels affected by *Sarcoptes scabiei* var*. cameli* [73], further supporting its involvement in host inflammatory responses to parasitic infestations. Our study also identified a protein cluster involving Cathelicidin, and S100A8/A12, suggesting that these molecules play an important role during the early stages of flystrike potentially to amplify inflammatory responses, consistent with previous observation in other parasites [74, 75].

Within the second cluster, A2M, FGB, SERPINF2 and HPX emerged as highly connected hub proteins, with A2M serving as the central seed. Notably A2M was also ranked among the top 10 hub proteins across the entire dataset. A2M is a well-known, broad spectrum, protease inhibitor that is involved in regulating proteolysis, although its physiological function has not yet been fully recognised [76]. Apart from being a protease inhibitor, it also binds with cytokines, toxins, bacterial and viral proteins and is considered an acute phase protein that is upregulated during inflammation [77]. Moreover, A2M also acts as a carrier of IL-6 and as a strong inducer of IL-6 synthesis [78]. During inflammatory responses, A2M provides protection against tissue damage by neutralizing proteases released from activated leukocytes, including neutrophils [79]. Previous studies have shown that flystrike lesions are characterised by neutrophil infiltration and elevated IL-6 and IL-8 levels [26, 28–30]. The detection of A2M in skin exudates therefore suggests that its secretion is a host response to skin injury and inflammation. Specifically, A2M may function to neutralize excessive protease activity, while also protecting IL-6 and IL-8 from proteolytic inactivation [80–82]. This is particularly relevant given previous observations of significantly increased local levels of IL-6 and IL-8 during flystrike [28–30]. Additionally, A2M has also been previously shown to significantly inhibit blowfly larval growth *in vivo* [33]. Thus, together with previous reports and our finding of A2M at the larval challenged site, soon after flystrike, suggest that A2M may contribute to controlling protease activity and modulating inflammation during early stages of flystrike.

The KEGG pathway enrichment analysis showed significant activation of complement and coagulation cascades and the NET formation pathway in larval-challenged sites. Indeed, wound exudates contained significantly high levels of fibrinogen, antithrombin III and complements including complement C2, complement C3 and complement C5. Additionally, complement components (i.e. C6, C7, factor B, factor I), which are involved in regulating the complement pathways, were also identified in the exudate collected from larval challenged sites. Supporting this finding, several proteins involved in complement pathways and coagulation cascade including fibrinogen and SERPINC1 (Antithrombin III) were also ranked in the top 10 hub protein list. Additionally, SERPINA1 (alfa-1 antitrypsin) was also detected as a top 10 hub protein indicating its extensive interaction with other proteins detected in an early flystrike lesion. It is an abundant plasma protein which increases following an infection or inflammation and can inhibit serine proteases including neutrophil elastases [83, 84]. Interestingly, it also has the potential to inhibit blowfly larval proteases and thereby inhibit the larval growth [33].

Another interesting finding in this study was the enrichment of the NET-formation pathway in flystrike lesions, where marked neutrophil infiltration has been reported [26, 27, 29, 30]. Neutrophils represent the first immunological barrier recruited to the site of infestation and deploy multiple effector mechanisms, including phagocytosis, degranulation and NETosis [85–87]. NETs are extracellular structures composed of nuclear DNA fibres decorated with histones, granule enzymes and antimicrobial peptides, which collectively act to immobilize and eliminate pathogens in the extracellular environment [88, 89]. Previous studies have demonstrated the involvement of NETs in controlling helminth parasites, such as *Strongyloides stercoralis* L3 larvae in a mouse model and *Haemonchus contortus* in bovine polymorphonuclear neutrophil cultures, where NETs effectively entrapped and reduced parasite viability [88, 90, 91]. While enrichment of the NET-formation pathway in early flystrike lesions is an interesting observation, particularly given evidence that NETosis can reduce the movement of nematodes, it is unclear whether this mechanism affects the much larger first instar blowfly larvae. While sheep produce various proteins to minimise tissue damage caused by both the inflammatory response and larval activity, blowfly larvae secrete proteolytic enzymes that degrade host tissues, creating a wound environment favourable to larval survival [92, 93]. Further integration of proteomic analyses of larval secretions with host responses could help determine whether these pathways represent genuine host defence mechanisms or are instead co-opted to facilitate parasite development.

This study compared the exudate collected from larval challenged sites of R with that of NS sheep and found no significant difference between the R and the NS flocks in the composition or intensity of the proteins detected in the exudates. These findings are consistent with our previous cellular and cytokine analyses in the same flocks, which also reported similar early immune responses between these same groups [30]. However, these findings were in contrast to the observation from the Trangie-resistant flock in which Complement C3 and α1 Antitrypsin levels were significantly different to that of the susceptible flock [35]. The selection of the Trangie flock was based upon selection of fleece-rot resistant sheep that also resulted in sheep that were less susceptible to flystrike [94]. By contrast, the flock that we used in the present study was exclusively selected on the basis of the field observation of sheep that had not been struck and possessed traits associated with breech strike resistance (i.e. less breech wrinkles). Given the difference in the selection approach in the R and NS sheep used in this study it is perhaps not surprising that differences in resistance mechanisms were observed.

Overall, our data suggest that the breech strike resistance traits that were being selected for in the Armidale sheep flock is not primarily driven by early immune or proteomic differences at the site of larval challenge. In our study, the time gap between blowfly egg deposition and larval collection was ~ 22 h. This comparatively short exposure period may have influenced the severity of the proteomic response, potentially contributing to the lack of significant differences detected between the resistant and susceptible sheep. Thus, longer exposure times may have resulted in more significant changes between the two populations of sheep. The selection of a short exposure was owing to our hypothesis that any meaningful protective host response would need to occur very early in the infection process before the larvae become well established [25, 95, 96] and was based on our hypothesis that the host response was key to controlling the larval challenge in the R sheep.

## Conclusions

Sheep mount a rapid local proteomic response to *Lucilia cuprina* infestation, characterised by activation of complement and coagulation pathways, NET formation and the involvement of key hub proteins such as Cathelicidin-2 and α-2-macroglobulin. However, no significant proteomic differences were detected between breech strike resistant and non-selected sheep at this early stage of larval infestation, suggesting that resistance in this flock is unlikely to be driven by early skin proteomic responses. These findings provide valuable insight into host–parasite interactions and a foundation for future investigations examining and comparing later-stage responses and alternative resistance mechanisms.

## Supplementary Information

Additional file1 (XLSX 27 KB)

Additional file2 (XLSX 11 KB)

Additional file3 (XLSX 32 KB)

Additional file4 (XLSX 20 KB)

Additional file5 (XLSX 17 KB)

Additional file6 (XLSX 51 KB)

## Data Availability

The mass spectrometry proteomics data have been deposited to the ProteomeXchange Consortium via the PRIDE [97, 98] partner repository with the dataset identifier PXD073234.
